# Blood python (*Python brongersmai*) strike kinematics and forces are robust to variations in substrate geometry

**DOI:** 10.1242/jeb.244456

**Published:** 2023-01-26

**Authors:** Derek J. Jurestovsky, Sidarth P. Joy, Henry C. Astley

**Affiliations:** ^1^ Department of Biology, University of Akron, 235 Carroll St, Akron, OH 44325, USA; ^2^ Biomechanics Laboratory, Pennsylvania State University, University Park, PA 16802, USA

**Keywords:** Rapid impulsive behaviors, Snake

## Abstract

Snake strikes are some of the most rapid accelerations in terrestrial vertebrates. Generating rapid body accelerations requires high ground reaction forces, but on flat surfaces snakes must rely on static friction to prevent slip. We hypothesize that snakes may be able to take advantage of structures in the environment to prevent their body from slipping, potentially allowing them to generate faster and more forceful strikes. To test this hypothesis, we captured high-speed video and forces from defensive strikes of juvenile blood pythons (*Python brongersmai*) on a platform that was either open on all sides or with two adjacent walls opposite the direction of the strike. Contrary to our predictions, snakes maintained high performance on open platforms by imparting rearward momentum to the posterior body and tail. This compensatory behavior increases robustness to changes in their strike conditions and could allow them to exploit variable environments.

## Teaser

**Highlighted Article:** Blood pythons maintain high strike performance on flat surfaces by imparting rearward momentum to the posterior body and tail, using them as an inertial appendage, allowing the snake to apply high fore–aft forces without slipping, and thus exploit variable environments.

## INTRODUCTION

Rapid impulsive behaviors (e.g. frog jumps, mantis shrimp strikes) are challenging for animals to produce, as they require high power and fast responses, and generate high forces on the body (Astley and Roberts, 2014; deVries et al., 2012; Ilton et al., 2018; Patek et al., 2004). Multiple factors can influence the power and acceleration of fast actions, including substrate rigidity (Astley et al., 2015; Demes et al., 1995), surface friction (Sutton and Burrows, 2008) and substrate geometry (Majumdar and Robergs, 2011). Predominately horizontal accelerations are particularly challenging to produce on flat substrates because slip will occur if the ratio between horizontal and vertical components exceeds the coefficient of friction (Hildebrand, 1989; Wilson et al., 2013). The surface's coefficient of friction determines the angle at which slip occurs (e.g. walking on ice versus asphalt) and thus how much lateral force an animal can apply (as a fraction of body weight) before losing its grip on the surface. In addition to friction, substrate geometry can greatly enhance an organism's ability to accelerate without losing static contact with a surface, by providing a rigid surface closer to perpendicular to the ground reaction force angle. For example, sprinters typically begin a race using starting blocks which provide them with inclined surfaces to apply propulsive force to, allowing them to apply higher horizontal forces without slipping (Majumdar and Robergs, 2011). The natural environment provides a wide range of variable substrate geometries which have the potential to affect an animal's performance of impulsive behaviors.

Striking snakes propel a large fraction of their anterior body forward with high accelerations (Herrel et al., 2011; Kardong, 1986; Kardong and Bels, 1998; Smith et al., 2002; Vincent et al., 2005; Young, 2010). Snakes are found on a wide range of substrates with highly variable surface friction and geometries, both of which will create challenges for striking. On flat, level, rigid ground a snake must rely on static friction to prevent slipping during mostly horizontal strikes (in which the forces are primarily oriented posteriorly to the strike direction with minimal lateral and vertical components), limiting how much force it can generate during a strike. Furthermore, while the low coefficient of friction between snake scales and the substrate is beneficial for locomotion, this exacerbates the problem of slip during striking (Baum et al., 2014; Benz et al., 2012). However, if the body can press against a rigid near-vertical surface (e.g. a rock, a log, etc.), a snake could potentially exert more force during its strike without slip.

**Fig. 1.**
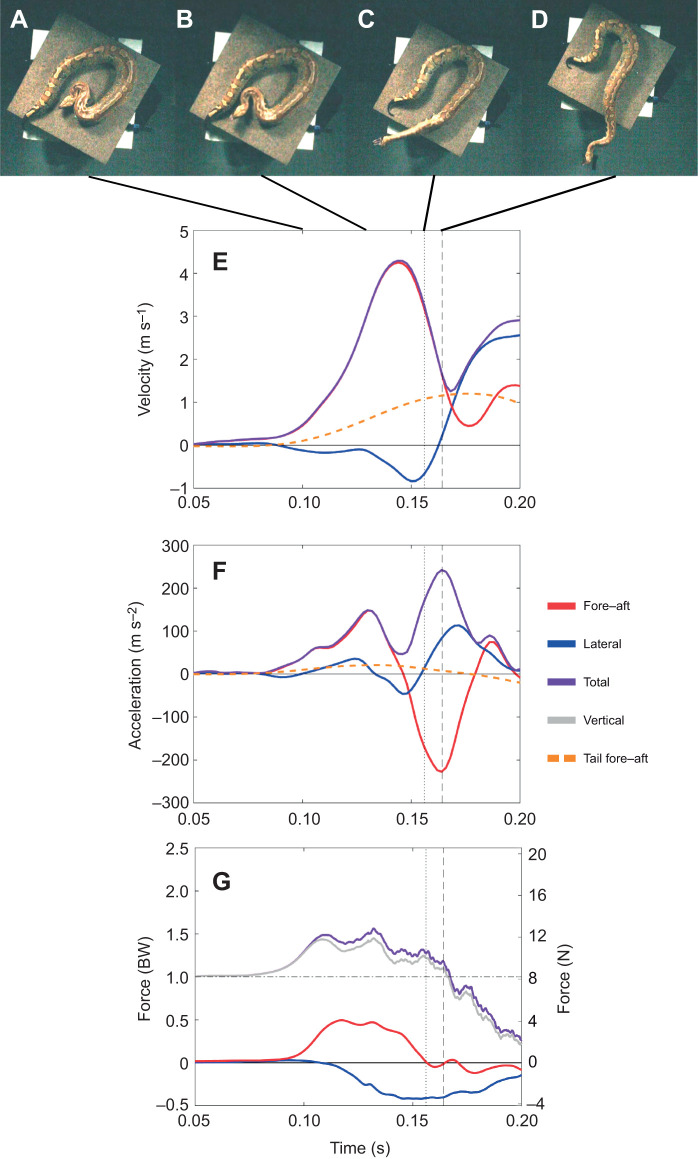
**Blood python strike in an open setup.** (A–D) Still images of the snake strike in the open setup at various stages of the strike including the beginning (A), point of maximum fore–aft force (B), point when the snake's neck is straight (C), and point when forward progress ends (D). (E–G) Corresponding graphs for the same strike of velocity (E), acceleration (F) and force (G) in body weights (BW) on the left and newtons (N) on the right. Solid red line is fore–aft force, solid blue line is lateral force, solid purple line is total force, solid gray line is vertical force and dashed orange line is tail fore–aft velocity/acceleration.

**Fig. 2.**
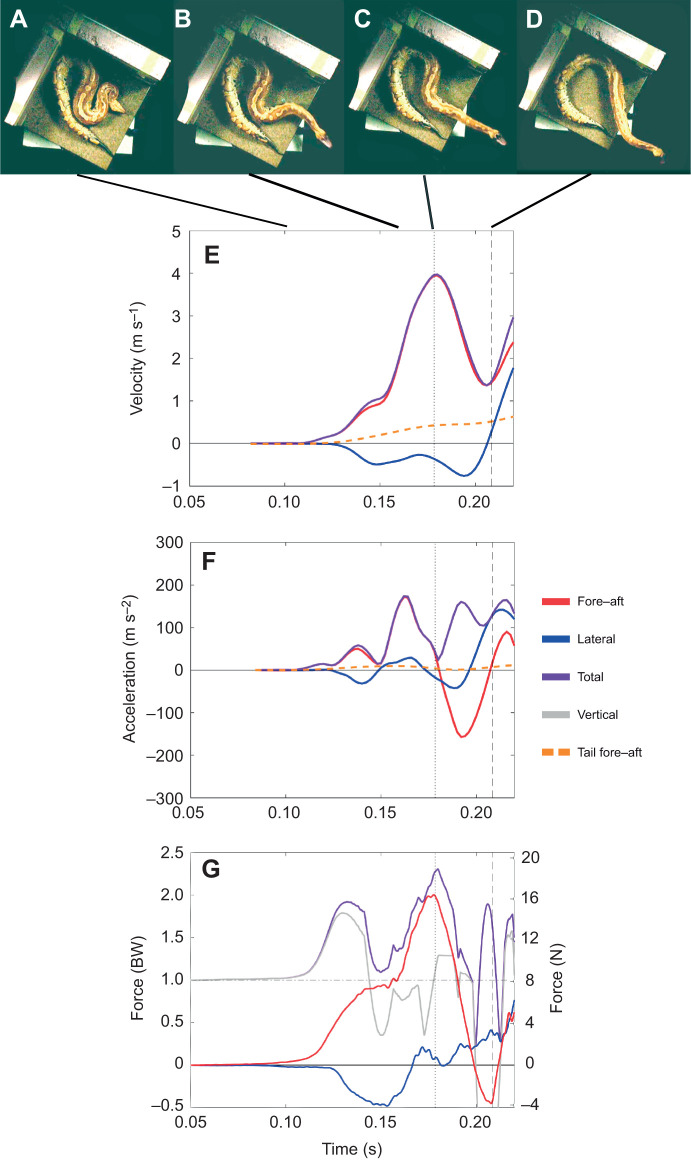
**Blood python strike in a walled setup.** (A–D) Still images of the snake strike in the walled setup at various stages of the strike including the beginning (A), point of maximum fore–aft force (B), point when the snake's neck is straight (C), and point when forward progress ends (D). (E–G) Corresponding graphs for the same strike of velocity (E), acceleration (F) and force (G) in body weights (BW) on the left and newtons (N) on the right. Solid red line is fore–aft force, solid blue line is lateral force, solid purple line is total force, solid gray line is vertical force and dashed orange line is tail fore–aft velocity/acceleration.

Snake strikes consistently show high head accelerations across taxa (56.8–199 m s^−2^) (Herrel et al., 2011; Kardong, 1975; Kardong and Bels, 1998; Moon et al., 2019; Penning et al., 2016; Ryerson and Tan, 2017; Ryerson and Van Valkenburg, 2021; Whitford et al., 2020). However, the substrate reaction forces that produce these rapid accelerations are unknown. We hypothesized that snakes will strike faster and with more force on a surface with vertically oriented features than on a featureless one. To test our hypothesis, we recorded synchronized kinematics and substrate reaction forces of the strikes for four blood pythons (*Python brongersmai*) on a custom-built platform with a high friction surface in two setups: a featureless plane and one with vertical walls that could serve as propulsive surfaces (Figs 1A–D and 2A–D).

## MATERIALS AND METHODS

Four wild-caught blood pythons, *Python brongersmai* Stull 1938, were obtained from a commercial provider [snout–vent length (SVL) mean±s.d. 76.1±6.2 cm, range 67.0–80.9 cm; mass 586.3±167.1 g, 450–670 g]. This species is highly suitable for strike studies because it is easily obtainable, non-venomous and strikes defensively with particular readiness. All experiments were approved by the University of Akron IACUC.

We constructed a rigid strike platform out of a 30.5×30.5×0.7 cm carbon fiber sandwich panel (DragonPlate, ALLRed & Associates Inc., Elbridge, NY, USA) covered in a rough material [Rock-on-a-Roll, Aquatica Water Gardens, Minneapolis, MN, USA; coefficient of friction (µ)=0.30±0.09]. The strike platform was attached to a six-axis force/torque sensor (Nano 43, ATI Industrial Automation, Apex, NC, USA), which was connected to a base made of expanded PVC board via two custom 3D printed ABS parts. Force data were collected using a NIDAQ N1-USB-6218 (16 bits, National Instruments, Austin, TX, USA) and recorded using the software IGOR Pro (Wavemetrics, Tigard, OR, USA) at 1 kHz. To dissuade the snakes from slithering off the platform, we raised it 83.0 cm off the ground using a frame of 80/20 supports and used clamps to attach the PVC board to the 80/20 supports anchored with sandbags. The walled setup was made by adding two adjacent walls made of rigid insulation foam attached to corrugated plastic board and screwed into the carbon fiber sheet on two adjacent sides. High-speed video was recorded at 500 images s^−1^ in dorsal view using an overhead SC1 Edgertronic high-speed camera (Sanstreak Corp., San Jose, CA, USA) 1.4 m above the strike platform. The trigger signal from the cameras was simultaneously recorded in IGOR via the NIDAQ, providing a method to synchronize the force and video recordings. Trials were performed in sets of three to five per 24 h and individuals were allowed a minimum of 5 min rest between trials to prevent fatigue. Snakes were not tested for 24 h after feeding occurred.

Strike trials were conducted after warming the snakes to 29–30°C, within the field active temperature of this species (Brattstrom, 1965, listed as *P. curtus*) using a modified insulated food container (Igloo Products Corp., Katy, TX, USA) with a heat cable (Zoo Med Laboratories Inc., San Luis Obispo, CA, USA) connected to a digital thermostat (Zoo Med Laboratories Inc.). We measured their temperature at three positions along the body using an eT650D Dual Laser Infrared Thermometer prior to testing (enno Logic, Eugene, OR, USA). After a snake was warmed and placed onto the strike platform, we induced strikes by moving side to side and/or quickly moving our hands to one side of the snake's head and back because one method failed to achieve strikes from all individuals. A total of 47 trials were recorded among the four individuals (24 for the open setup followed by 23 for the walled setup) with five to seven trials per individual per setup. We digitized the locations of the heads and tails of our snakes using the MATLAB application DLTdv8a (Hedrick, 2008). Next, we used coordinate transformation to reorient both the force sensor axes and the axes of the digitization to align with the strike direction (defined as the overall direction of the snake's head movement after 10 frames from when the strike began) using the two equations:
(1)

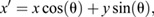

(2)

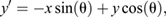
where θ is the angle between the original axis (i.e. force sensor or digitization) and the new axis (direction of the snake strike). Force and kinematic data were splined in order to smooth data over time and avoid end effects seen when filtering non-cyclic data, then processed using a custom-written MATLAB script (MathWorks, Natick, MA, USA) (Supplementary Materials and Methods). We measured 13 variables: maximum fore–aft force, maximum lateral force, maximum vertical force, maximum total force, maximum head velocity, maximum head acceleration, fore–aft impulse (the integral of fore–aft force from the beginning of the strike until forward progress ends), strike distance, maximum tail velocity in the strike direction, maximum tail acceleration in the strike direction, maximum tail displacement in the strike direction, maximum fore–aft to vertical force (FA/V) ratio, and the percentage of the strike above the slip threshold (a FA/V ratio above the substrate coefficient of friction, µ=0.3). Forces obtained from the sensor were divided by the snake mass×gravity, which is a unitless variable, and is reported in body weights (BW). Because of multiple snakes leaving the camera view in the walled setup, we removed strike duration from our analysis but retained the data within Table 1 for comparison. Maximum values (e.g. maximum force and velocity) were calculated using the MATLAB peak function. Impulse was calculated using the MATLAB trapz function.

**Table 1.**
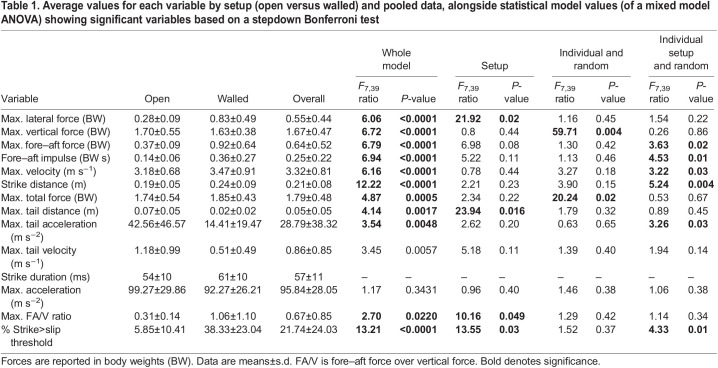
Average values for each variable by setup (open versus walled) and pooled data, alongside statistical model values (of a mixed model ANOVA) showing significant variables based on a stepdown Bonferroni test

To test whether variables differ between the open and walled setups, we ran a mixed model ANOVA for each variable with setup, individual, and setup×individual as factors with a random effect assigned to both the individual and setup×individual, implemented in JMP Pro 15 (SAS Institute Inc., Cary, NC, USA). We then used the Brown–Forsythe modification of Levene's test to determine whether individual snakes showed greater variation in one setup than the other for all variables under consideration (Brown and Forsythe, 1974; Levene, 1961). These tests were implemented in R 3.6.0, package *car* (Fox and Weisberg, 2018; https://CRAN.R-project.org/package=car). To correct for multiple comparisons in the test for homogeneity of variance, we used the Bonferroni–Holm step-down procedure.

## RESULTS AND DISCUSSION

Snakes displayed high strike performance in both setups, with maximum fore–aft force of 0.64±0.52 BW, maximum total force of 1.79±0.48 BW, maximum head velocity of 3.32±0.81 m s^−1^, maximum head acceleration of 95.84±28.05 m s^−2^, strike distance of 0.21±0.08 m, maximum FA/V ratio of 0.67±0.85, percentage of the strike above the slip threshold of 21.74±24.03% and strike duration of 57±11 ms (Table 1; see Tables S1 for all values). Our results displayed high individual variability within and between individuals and setups. As a result, the open and walled setups were statistically indistinguishable in most variables measured (Table 1; see Table S1 for all values; Figs 1 and 2). Only maximum lateral force, maximum tail distance, maximum FA/V ratio and percentage of the strike above the slip threshold were significantly affected by the setup (Table 1). Lateral force was significantly higher in the walled setup, while the tail moved a greater distance in the open setup (Table 1). As we hypothesized, the maximum ratio of fore–aft to vertical force was much lower in the open setup compared with the walled setup, and exceeded the coefficient of friction for a much smaller fraction of the strike (Table 1). Individual and individual×setup effects were common, emphasizing the high variability of striking behavior (Table 1). ANOVA effect estimates and confidence intervals are given in (Table S3). The Levene's test (using the Brown–Forsythe modification) showed that variance was similar between individuals and setups for all variables (Table S2).

We recorded velocities and accelerations (Table 1) similar to previously reported values in various snake taxa (Ryerson and Van Valkenburg, 2021, see their table 4 and references therein), further supporting previous studies showing snakes across a variety of families and body forms strike with similarly fast kinematics (Ryerson and Van Valkenburg, 2021). In addition to recording kinematic data, our study is the first to record ground reaction forces during snake strikes (Figs 1 and 2, Table 1). The blood pythons in our study generated almost 1.0 BW horizontally and up to 1.7 BW vertically (Fig. 2). Our results show striking similarities to the ground reaction forces measured during a human punch (just below 0.5 BW in both the fore–aft and lateral directions and roughly 1.5 BW in vertical force) (Lenetsky et al., 2020). There is support for the role of trunk rotation converting vertical ground reaction force to horizontal force during a punch (Tong-Iam et al., 2017), and another study showed fatiguing lower-body exercise diminished punch performance, highlighting the importance of the ground reaction forces generated during a punch (Dunn et al., 2022). Future work exploring the ground reaction forces in other systems with rapid movement of body parts, such as chameleon tongue projection and heron predatory strikes, would provide a stronger basis for broad, comparative conclusions.

Strike kinematics have been measured in a variety of snakes (Herrel et al., 2011; Penning et al., 2016, 2020; Ryerson and Tan, 2017; Ryerson and Van Valkenburg, 2021; Whitford et al., 2020; Young, 2010). However, the kinetics applied to the environment are essential to understanding the kinematics of the snake's head. In order to impart momentum to the head of the snake, equal and opposite momentum must be imparted to the ground, the tail or a combination thereof. The force sensor detects only the momentum imparted to the ground; thus, if there is momentum imparted in the opposite direction by the movement of the tail, the impulse computed from the ground reaction force will provide a lower value than the true momentum imparted to the head. In contrast, if the snake is backed against a solid substrate and the tail moves little, then ground reaction forces will accurately capture forces acting on the anterior body. Our results match what would be expected if the tail and posterior body were being used as an inertial appendage. As stated above, slip occurs when the ratio of fore–aft to vertical force is greater than the coefficient of friction (µ=0.30 in our system). In the open setup, the maximum FA/V ratio of 0.31±0.14 is just above the coefficient of friction and the ratio exceeds 0.30 for only 5.85±10.41% of the strike, whereas in the walled setup the ratio is 1.06±1.10, well above the coefficient of friction, and these values are maintained for 38.33±23.04% of the strike (Table 1). This suggests that in the open setup, the movement of the tail is keeping the snakes' FA/V ratio at or just below the coefficient of friction, preventing slip and allowing the snake to achieve similar velocities to those in the walled setup. Furthermore, blood pythons show a similar chronic retention of fecal mass to that seen in other heavy-bodied snakes, which has been hypothesized to serve as inert ballast to maximum body inertia and friction with the substrate during striking (Lillywhite et al., 2002). This posteriorly located fecal mass would also be beneficial to the active use of the tail and posterior body as an inertial appendage.

We expect this inertial mechanism is not limited to blood pythons and could be exploited by multiple other snake taxa. Specifically, this mechanism is likely to be exploited in similarly large-bodied snake taxa where the posterior of the body is robust in size. As such, we expect this mechanism to be less likely in more gracile snakes such as colubrids where their tails are relatively thin by comparison. A large variety of snakes will encounter open habitats and this mechanism would enable them to strike with similar performance to when they are backed against a wall, partially buried, gripping a branch, etc., allowing them to exploit a wider range of microhabitats to successfully capture prey. Few studies analyze behavior in the wild of non-vipers. Only a handful of studies on foraging in vipers describe the ambush sites; however, they are typically listed as under a bush, in thick or thin vegetation, or partial burial (Barbour and Clark, 2012; Clark et al., 2016; Horesh et al., 2017). Even fewer studies analyze foraging behavior in non-vipers, none of which offer a detailed description of the ambush sites. Furthermore, the sites described as ambush locations are ambiguous in regard to whether the snake is backed against foliage or simply under the cover of the foliage. Only partial burial specifically describes a situation where the snake is backed against a structure. However, it is relatively unlikely that in every such case, the snake is completely backed against a structure and it is possible the mechanism of the tail as an inertial appendage could be employed. As such, future studies analyzing foraging behavior in snakes should attempt to discern whether the snakes in ambush are backed against a structure or not when possible. The use of the tail as an inertial appendage has been studied in geckos, cheetahs, monkeys and squirrels, showing these animals using their tails as an inertial appendage for a variety of behaviors including balance, to reorient themselves mid-air, and for faster, tighter turns (Fukushima et al., 2021; Jusufi et al., 2010; Patel and Braae, 2013; Young et al., 2015). However, quantifying the momentum transfer of the continuous body of these blood pythons in frictional contact with the substrate is beyond the scope of our study. The ability to achieve similar performance in a variety of substrates and settings could have multiple benefits for a species and could be a contributing factor to the success of snakes in exploiting a diverse array of habitats.

## Supplementary Material

10.1242/jexbio.244456_sup1 Supplementary information
