# Supplementary material for: Blood python (Python brongersmai) strike kinematics and forces are robust to variations in substrate geometry
Source: J Exp Biol. 2023 Jan 26;226(2):jeb244456. doi: 10.1242/jeb.244456 (PMC10086539; doi:10.1242/jeb.244456)
Supplement: Supplementary information [file jexbio-226-244456-s1.pdf]

Table S1. Snake strike data from the open and walled setups for all individuals. Superscript a denotes the open setup and superscript b denotes the walled setup. Forces are reported in body weights (BW). FA/V is fore-aft force over vertical force. Avg. = average, accel. = acceleration, dist. = distance, dur. = duration, ind. = individual, lat. = lateral, max. = maximum, str. = strike, thresh. = threshold, vel. = velocity, and vert. = vertical.

Ind.	Trial	Max. Fore-aft Force (BW)	Max. Lat. Force (BW)	Max. Vert. Force (BW)	Max. Total Force (BW)	Fore-Aft Impulse (BW*s)	Max. Vel. (m/s)	Max. Accel. (m/s^2)	Str. Dist. (m)	Max. Tail Vel.	Max. Tail Accel. (m/s^2)	Str. Dur. (ms)	Max. Tail Dist. (m)	Max. FA/V Ratio	% Strike > Slip Thresh.
1[a]	1	0.33	0.14	1.58	1.60	0.15	2.48	80.16	0.22	0.73	31.48	48	0.03	0.29	0
	2	0.51	0.30	1.80	1.88	0.25	2.88	104.02	0.24	1.98	49.39	44	0.14	0.29	0
	3	0.41	0.17	3.74	3.76	0.05	3.38	119.90	0.14	1.28	28.31	58	0.06	0.23	0
	4	0.32	0.28	1.70	1.73	0.06	3.77	121.95	0.15	0.62	9.33	56	0.06	0.29	0
	5	0.51	0.33	1.86	1.94	0.21	2.20	77.18	0.16	0.67	18.42	46	0.04	0.31	13
	6	0.32	0.27	1.75	1.78	0.13	2.82	104.73	0.17	1.07	31.21	54	0.10	0.25	0
3[a]	1	0.20	0.30	1.25	1.28	0.06	3.82	157.56	0.23	1.25	28.45	54	0.08	0.34	22
	2	0.42	0.35	1.03	1.14	0.19	4.26	148.62	0.18	1.20	31.30	44	0.10	0.13	0
	3	0.50	0.39	1.05	1.09	0.19	3.82	95.32	0.19	0.67	25.73	70	0.03	0.80	35
	4	0.39	0.37	1.27	1.45	0.12	4.50	122.64	0.21	0.91	46.92	68	0.07	0.39	20
	5	0.41	0.15	1.06	1.12	0.20	3.66	102.56	0.19	0.84	23.73	40	0.09	0.48	28
5[a]	1	0.44	0.25	1.56	1.62	0.14	3.08	96.37	0.12	1.30	62.80	42	0.09	0.17	0
	2	0.34	0.31	1.64	1.65	0.13	3.63	118.40	0.21	0.55	14.24	58	0.03	0.50	16
	3	0.34	0.16	1.41	1.46	0.14	3.23	114.48	0.14	0.17	4.00	48	0.01	0.27	0
	4	0.25	0.15	1.59	1.60	0.11	2.54	48.89	0.15	1.66	59.27	66	0.09	0.20	0
	5	0.34	0.20	1.59	1.59	0.12	3.37	111.41	0.15	0.40	8.22	58	0.04	0.26	0
	6	0.37	0.35	1.43	1.50	0.16	3.75	136.83	0.16	0.61	25.14	70	0.06	0.30	0
	7	0.20	0.21	1.36	1.38	0.09	2.05	36.74	0.08	0.23	5.28	68	0.02	0.15	0
6[a]	1	0.39	0.22	1.86	1.89	0.12	3.24	89.31	0.24	1.82	101.63	54	0.07	0.29	0
	2	0.32	0.39	2.03	2.03	0.13	3.49	108.69	0.25	0.69	21.46	54	0.05	0.24	0
	3	0.32	0.44	2.13	2.14	0.17	2.37	72.08	0.29	1.09	18.74	50	0.03	0.26	0
	4	0.31	0.37	2.01	2.04	0.08	3.19	86.09	0.32	3.89	161.88	52	0.22	0.31	2
	5	0.50	0.17	1.98	2.03	0.31	2.16	70.91	0.21	4.15	190.20	32	0.19	0.29	0

	6	0.43	0.35	2.17	2.20	0.16	2.53	57.59	0.23	0.64	24.31	52	0.03	0.32	5
Avg.[a]		0.37±0.09	0.28±0.09	1.70±0.55	1.74±0.54	0.14±0.06	3.18±0.68	99.27±29.86	0.19±0.05	1.18±0.99	42.56±46.57	54±10	0.07±0.05	0.31±0.14	6±10
1[b]	1	1.55	0.98	1.99	2.01	0.41	3.55	78.91	0.31	0.09	2.08	78	0.03	3.02	58
	2	1.96	1.58	2.19	2.57	0.72	3.56	138.22	0.36	2.16	90.93	54	0.05	2.14	19
	3	1.86	1.39	1.84	1.94	0.84	4.68	68.29	0.37	0.64	27.69	64	0.007	0.84	39
	4	0.76	1.06	1.51	1.86	0.31	3.42	103.11	0.18	0.49	7.48	60	0.02	0.64	34
	5	0.70	0.58	2.02	2.20	0.89	4.83	81.28	0.31	0.65	6.38	94	0.09	0.32	63
3[b]	1	1.70	0.94	0.99	1.98	0.50	3.96	74.85	0.22	0.68	9.61	72	0.02	4.20	60
	2	1.71	0.41	1.32	1.86	0.73	3.95	58.34	0.23	0.16	21.53	60	0.01	1.31	34
	3	0.37	0.14	1.31	1.31	0.20	3.80	129.98	0.27	0.74	9.81	52	0.01	0.44	58
	4	0.49	0.58	1.26	1.29	0.16	5.74	72.00	0.32	0.66	23.85	62	0.003	0.52	33
	5	0.38	1.32	1.09	1.51	0.15	4.13	83.16	0.28	0.91	38.96	74	0.05	0.37	17
	6	0.32	0.88	1.32	1.55	0.22	3.87	147.96	0.23	0.15	6.36	64	0.001	1.04	70
5[b]	1	0.35	0.92	1.38	1.56	0.17	3.14	84.86	0.17	0.25	7.06	46	0.02	0.27	0
	2	0.27	0.17	1.54	1.54	0.11	2.99	102.84	0.12	0.15	7.54	54	0.01	0.21	0
	3	0.20	0.70	1.24	1.37	0.09	2.40	90.75	0.10	0.22	4.14	62	0.01	0.24	0
	4	0.68	0.46	2.09	2.17	0.28	2.88	97.10	0.16	0.49	13.00	54	0.02	0.58	59
	5	0.29	0.78	1.34	1.34	0.06	2.58	108.00	0.10	0.12	1.63	68	0.004	0.33	9
	6	0.33	0.43	1.38	1.43	0.14	1.82	58.82	0.08	0.17	3.60	56	0.003	0.44	19
6[b]	1	1.38	0.33	1.61	1.69	0.83	3.49	70.64	0.20	1.28	20.88	62	0.02	3.31	67
	2	1.14	1.88	2.02	2.08	0.27	2.33	94.28	0.26	0.79	20.46	48	0.03	0.94	65
	3	1.45	1.51	2.21	2.43	0.31	4.22	138.29	0.37	0.03	0.02	58	0.004	1.54	42
	4	2.04	0.79	2.05	2.93	0.53	3.32	104.89	0.33	0.26	2.60	54	0.03	0.82	48
	5	0.52	0.13	1.88	1.91	0.25	2.59	63.71	0.23	0.62	5.48	56	0.03	0.33	34
	6	0.59	1.22	1.89	1.91	0.12	2.63	71.88	0.30	0.06	0.20	52	0.01	0.43	54
Avg.[b]		0.92±0.64	0.83±0.49	1.63±0.38	1.85±0.43	0.36±0.27	3.47±0.91	92.27±26.21	0.24±0.09	0.51±0.49	14.41±19.47	61±11	0.02±0.02	1.06±1.09	38±23

Journal of Experimental Biology • Supplementary information

Table S2. Brown-Forsythe modification of Levene's test showing variation per individual, setup, and variable. Note that none of the variables are significant. Forces are reported in body weights (BW). FA/V is fore-aft force over vertical force. Adj. = adjusted, deg. = degrees, dev. = deviation, max. = maximum, and std. = standard.

Variable	Individual	Deg. of Freedom	F-value	p-adj.	Std. Dev. Open	Std. Dev. Walled
Max. Fore-aft Force (BW)	1	1,9	7.487	0.989	0.091	0.600
	3	1,9	2.116	1	0.109	0.682
	5	1,11	0.791	1	0.076	0.167
	6	1,10	8.019	0.801	0.074	0.571
Max. Lateral Force (BW)	1	1,9	5.365	1	0.075	0.389
	3	1,9	7.536	0.997	0.095	0.418
	5	1,11	10.557	0.356	0.076	0.273
	6	1,10	14.630	0.161	0.105	0.682
Max. Vertical Force (BW)	1	1,9	0.390	1	0.821	0.253
	3	1,9	0.044	1	0.118	0.140
	5	1,11	0.613	1	0.111	0.306
	6	1,10	1.581	1	0.111	0.201
Max. Total Force (BW)	1	1,9	0.399	1	0.818	0.282
	3	1,9	1.341	1	0.149	0.280
	5	1,11	1.365	1	0.097	0.305
	6	1,10	3.268	1	0.107	0.451
Fore-aft Impulse (BW)	1	1,9	4.238	1	0.078	0.261
	3	1,9	1.464	1	0.060	0.235
	5	1,11	3.011	1	0.024	0.077
	6	1,10	1.886	1	0.078	0.256
Max. Velocity (m/s)	1	1,9	0.076	1	0.580	0.687
	3	1,9	0.109	1	0.355	0.741
	5	1,11	0.131	1	0.608	0.483
	6	1,10	0.437	1	0.545	0.711

Journal of Experimental Biology • Supplementary information

Max. Acceleration (m/s^2)	1	1,9	0.188	1	19.081	27.788
	3	1,9	0.102	1	27.420	35.901
	5	1,11	1.237	1	37.569	17.536
	6	1,10	1.009	1	17.839	28.157
Strike Distance (m)	1	1,9	0.583	1	0.039	0.077
	3	1,9	2.387	1	0.019	0.040
	5	1,11	0.042	1	0.039	0.037
	6	1,10	1.939	1	0.039	0.063
Max. Tail Velocity (m/s)	1	1,9	0.041	1	0.520	0.790
	3	1,9	0.099	1	0.246	0.318
	5	1,11	3.311	1	0.563	0.134
	6	1,10	3.753	1	1.588	0.486
Max. Tail Acceleration (m/s^2)	1	1,9	0.734	1	13.567	37.131
	3	1,9	0.889	1	9.225	12.314
	5	1,11	3.630	1	25.260	4.016
	6	1,10	12.977	0.227	76.621	9.775
Max. Tail Distance (m)	1	1,9	0.114	1	0.039	0.032
	3	1,9	0.456	1	0.027	0.019
	5	1,11	5.916	1	0.032	0.007
	6	1,10	3.653	1	0.084	0.010
Max. FA/V Ratio	1	1,9	5.113	1	0.030	1.143
	3	1,9	1.544	1	0.244	1.462
	5	1,11	0.291	1	0.118	0.141
	6	1,10	3.556	1	0.030	1.107
% Strike > Slip Threshold	1	1,9	5.551	1	5.307	18.008
	3	1,9	3.170	1	13.115	20.334
	5	1,11	2.389	1	6.047	23.072
	6	1,10	11.450	0.376	2.041	12.941

Table S3. 95% confidence intervals for all variables and individuals by setup. The walled setup is the base category for setup and individual six is the base category for individual. Bold denotes significance. Forces are reported in body weights (BW). FA/V is fore-aft force over vertical force. Max. = maximum, dist. = distance, std. = standard, and thresh. = threshold.

Variable	Term	Estimate	Std. Error	t Ratio	p-value	Lower 95%	Upper 95%
Max. Fore-Aft Force (BW)	Intercept	0.653	0.056	11.66	**<.0001**	0.540	0.767
	Setup[open]	-0.281	0.056	-5.03	**<.0001**	-0.395	-0.168
	Individual[1]	0.231	0.099	2.33	**0.0252**	0.030	0.431
	Individual[3]	-0.048	0.099	-0.48	0.633	-0.248	0.153
	Individual[5]	-0.313	0.094	-3.34	**0.002**	-0.502	-0.123
	Individual[1]*Setup[open]	-0.202	0.099	-2.04	**0.0478**	-0.403	-0.002
	Individual[3]*Setup[open]	0.059	0.099	0.59	0.557	-0.142	0.259
	Individual[5]*Setup[open]	0.267	0.094	2.85	**0.007**	0.078	0.457
Max. Lateral Force (BW)	Intercept	0.562	0.049	11.5	**<.0001**	0.463	0.661
	Setup[open]	-0.284	0.049	-5.8	**<.0001**	-0.382	-0.185
	Individual[1]	0.121	0.087	1.4	0.171	-0.054	0.296
	Individual[3]	-0.051	0.087	-0.59	0.561	-0.226	0.124
	Individual[5]	-0.157	0.082	-1.92	0.063	-0.322	0.009
	Individual[1]*Setup[open]	-0.152	0.087	-1.75	0.087	-0.327	0.023
	Individual[3]*Setup[open]	0.084	0.087	0.97	0.337	-0.091	0.259
	Individual[5]*Setup[open]	0.112	0.082	1.37	0.180	-0.054	0.277
Max. Vertical Force (BW)	Intercept	1.663	0.050	33.08	**<.0001**	1.562	1.765
	Setup[open]	0.023	0.050	0.45	0.652	-0.079	0.125
	Individual[1]	0.327	0.089	3.67	**0.001**	0.147	0.507
	Individual[3]	-0.489	0.089	-5.5	**<.0001**	-0.669	-0.310
	Individual[5]	-0.161	0.084	-1.92	0.063	-0.331	0.009
	Individual[1]*Setup[open]	0.059	0.089	0.66	0.513	-0.121	0.239
	Individual[3]*Setup[open]	-0.064	0.089	-0.72	0.476	-0.244	0.116
	Individual[5]*Setup[open]	-0.015	0.084	-0.18	0.861	-0.185	0.155

Max. Total Force (BW)	Intercept	1.794	0.056	31.89	**<.0001**	1.680	1.908
	Setup[open]	-0.063	0.056	-1.11	0.273	-0.176	0.051
	Individual[1]	0.321	0.100	3.22	**0.003**	0.119	0.522
	Individual[3]	-0.394	0.100	-3.96	**0.000**	-0.595	-0.193
	Individual[5]	-0.239	0.094	-2.54	**0.015**	-0.429	-0.048
	Individual[1]*Setup[open]	0.061	0.100	0.62	0.542	-0.140	0.263
	Individual[3]*Setup[open]	-0.121	0.100	-1.22	0.232	-0.322	0.080
	Individual[5]*Setup[open]	0.049	0.094	0.52	0.603	-0.141	0.240
Fore-Aft Impulse (BW)	Intercept	0.259	0.023	11.12	**<.0001**	0.212	0.306
	Setup[open]	-0.113	0.023	-4.86	**<.0001**	-0.160	-0.066
	Individual[1]	0.129	0.041	3.13	**0.003**	0.046	0.213
	Individual[3]	-0.020	0.041	-0.48	0.635	-0.103	0.064
	Individual[5]	-0.125	0.039	-3.2	**0.003**	-0.204	-0.046
	Individual[1]*Setup[open]	-0.133	0.041	-3.23	**0.003**	-0.217	-0.050
	Individual[3]*Setup[open]	0.026	0.041	0.63	0.533	-0.057	0.109
	Individual[5]*Setup[open]	0.105	0.039	2.7	**0.010**	0.026	0.184
Max. Velocity (m/s)	Intercept	3.354	0.089	37.88	**<.0001**	3.175	3.533
	Setup[open]	-0.140	0.089	-1.58	0.122	-0.319	0.039
	Individual[1]	0.109	0.157	0.7	0.491	-0.208	0.426
	Individual[3]	0.772	0.157	4.93	**<.0001**	0.456	1.089
	Individual[5]	-0.491	0.148	-3.31	**0.002**	-0.790	-0.191
	Individual[1]*Setup[open]	-0.403	0.157	-2.57	**0.014**	-0.720	-0.086
	Individual[3]*Setup[open]	0.027	0.157	0.17	0.867	-0.290	0.343
	Individual[5]*Setup[open]	0.369	0.148	2.49	**0.017**	0.069	0.669
Max. Acceleration (m/s^2)	Intercept	96.440	4.062	23.74	**<.0001**	88.225	104.656
	Setup[open]	4.102	4.062	1.01	0.319	-4.114	12.318
	Individual[1]	1.202	7.188	0.17	0.868	-13.337	15.740
	Individual[3]	13.419	7.188	1.87	0.069	-1.119	27.958
	Individual[5]	-3.877	6.796	-0.57	0.572	-17.623	9.868

Journal of Experimental Biology • Supplementary information

	Individual[1]*Setup[open]	-0.422	7.188	-0.06	0.954	-14.961	14.116
	Individual[3]*Setup[open]	11.377	7.188	1.58	0.122	-3.162	25.915
	Individual[5]*Setup[open]	-1.933	6.796	-0.28	0.778	-15.679	11.812
Strike Distance (m)	Intercept	21.749	0.682	31.87	**<.0001**	0.204	0.231
	Setup[open]	-2.321	0.682	-3.4	**0.002**	-0.037	-0.009
	Individual[1]	2.340	1.207	1.94	0.060	-0.001	0.048
	Individual[3]	1.068	1.207	0.88	0.382	-0.014	0.035
	Individual[5]	-8.543	1.142	-7.48	**<.0001**	-0.109	-0.062
	Individual[1]*Setup[open]	-3.951	1.207	-3.27	**0.002**	-0.064	-0.015
	Individual[3]*Setup[open]	-0.596	1.207	-0.49	0.624	-0.030	0.018
	Individual[5]*Setup[open]	3.443	1.142	3.02	**0.005**	0.011	0.058
Max. Tail Velocity (m/s)	Intercept	1.046	0.095	11	**<.0001**	0.853	1.238
	Setup[open]	0.337	0.095	3.55	**0.001**	0.145	0.530
	Individual[1]	0.175	0.168	1.04	0.305	-0.165	0.515
	Individual[3]	0.062	0.168	0.37	0.713	-0.278	0.403
	Individual[5]	-0.458	0.159	-2.88	**0.006**	-0.780	-0.137
	Individual[1]*Setup[open]	0.054	0.168	0.32	0.751	-0.286	0.394
	Individual[3]*Setup[open]	-0.201	0.168	-1.19	0.240	-0.541	0.140
	Individual[5]*Setup[open]	-0.047	0.159	-0.3	0.767	-0.369	0.274
Max. Tail Acceleration (m/s^2)	Intercept	31.839	4.175	7.63	**<.0001**	23.394	40.284
	Setup[open]	11.414	4.175	2.73	**0.009**	2.969	19.859
	Individual[1]	4.556	7.388	0.62	0.541	-10.388	19.501
	Individual[3]	2.022	7.388	0.27	0.786	-12.922	16.967
	Individual[5]	-14.247	6.986	-2.04	**0.048**	-28.376	-0.117
	Individual[1]*Setup[open]	-0.634	7.388	-0.09	0.932	-15.579	14.310
	Individual[3]*Setup[open]	-3.624	7.388	-0.49	0.627	-18.568	11.321
	Individual[5]*Setup[open]	-1.689	6.986	-0.24	0.810	-15.818	12.441
Max. Tail Distance (m)	Intercept	7.474	0.677	11.04	**<.0001**	0.061	0.088
	Setup[open]	2.934	0.677	4.33	**<.0001**	0.016	0.043

	Individual[1]	1.257	1.198	1.05	0.300	-0.012	0.037
	Individual[3]	0.578	1.198	0.48	0.632	-0.018	0.030
	Individual[5]	-3.051	1.133	-2.69	**0.010**	-0.053	-0.008
	Individual[1]*Setup[open]	-0.814	1.198	-0.68	0.501	-0.032	0.016
	Individual[3]*Setup[open]	-0.549	1.198	-0.46	0.649	-0.030	0.019
	Individual[5]*Setup[open]	-0.033	1.133	-0.03	0.977	-0.023	0.023
Max. FA/V Ratio	Intercept	0.692	0.111	6.22	**<.0001**	0.467	0.917
	Setup[open]	-0.379	0.111	-3.41	**0.0015**	-0.604	-0.154
	Individual[1]	0.143	0.197	0.73	0.471	-0.255	0.541
	Individual[3]	0.180	0.197	0.91	0.366	-0.218	0.578
	Individual[5]	-0.386	0.186	-2.08	**0.045**	-0.763	-0.010
	Individual[1]*Setup[open]	-0.179	0.197	-0.91	0.368	-0.578	0.219
	Individual[3]*Setup[open]	-0.065	0.197	-0.33	0.742	-0.463	0.333
	Individual[5]*Setup[open]	0.338	0.186	1.82	0.077	-0.038	0.715
% Strike > Slip Thresh.	Intercept	22.563	2.085	10.82	**<.0001**	18.347	26.780
	Setup[open]	-15.941	2.085	-7.65	**<.0001**	-20.158	-11.725
	Individual[1]	-0.240	3.689	-0.07	0.949	-7.701	7.222
	Individual[3]	10.620	3.689	2.88	**0.0064**	3.159	18.082
	Individual[5]	-14.174	3.488	-4.06	**0.0002**	-21.229	-7.120
	Individual[1]*Setup[open]	-4.208	3.689	-1.14	0.261	-11.669	3.254
	Individual[3]*Setup[open]	3.671	3.689	1	0.326	-3.790	11.133
	Individual[5]*Setup[open]	9.831	3.488	2.82	**0.0075**	2.776	16.885

Supplementary Materials and Methods. Code used to process and analyze data.

```
clear all

data1 = dlmread('S3T7_7_7_21bpxypts.csv',',',1,0); %1 skips rows(top down) and skips 0 columns (left to right)
dataa = dlmread('S3T7_7_7_21.csv',',',1,0); %1 skips rows(top down) and skips 0 columns (left to right)
datam = dlmread('S3T7_7_7_21bptxypts.csv',',',1,0); %1 skips rows(top down) and skips 0 columns (left to right)

data2=data1;
datab=dataa;
datan=datam;

%Data 1 Manipulations:
%Load data
time = data2(:,[1]);
x = data2(:,[2]);
y = data2(:,[3]);
framerate = 500;

%Adjust values to 'zero' them
x1 = x-x(30);
y1 = y-y(30);

%Convert pixels to mm
x2 = x1/1.15;
y2 = y1/1.15;

%shift x and y axis to align with force sensor axis
xx = x2*cos(37)+y2*sin(37);
yy = -x2*sin(37)+y2*cos(37);

%SMOOTH BEFORE CALCULATING
[sp,sxx]=spaps(time,xx,0.5); %0.5 looked good
```

Journal of Experimental Biology • Supplementary information

```
[sp,syy]=spaps(time,yy,0.5); %0.5 looked good


Vx=diff(sxx)*framerate; %where framerate = 500.  If framerate is 1/500, use diff(x2)/framerate
Vy=diff(syy)*framerate;
Vxy=sqrt((Vx.^2)+(Vy.^2));

%Set 0
zero1=Vx*0;
timez = time*1;
%Get hypotenuse
h = sqrt((sxx.^2)+(syy.^2));

%Take derivative of position data revealing velocity
% vel = gradient(y2(:))./gradient(x2(:));
%
% vel1 = gradient(h(:))./gradient(time(:));
%
% vel2 = vel1/1000;

%Data a manipulations:
%Load data
timea = datab(:,[1]);
fx = datab(:,[2]);
fy = datab(:,[3]);
fz = datab(:,[4]);

%Zero time/forces
timeb = timea-timea(1);
fx1 = fx-fx(1);
fy1 = fy-fy(1);
fz1 = fz-fz(1);

%Get hypotenuse
```

Journal of Experimental Biology • Supplementary information

```
ha = sqrt((fx1.^2)+(fy1.^2)+(fz1.^2));



%convert mm to m
Vx1=Vx/1000;
Vy1=Vy/1000;
Vxy1=Vxy/1000;
zero2 = timeb*0;


Forceint = trapz(ha(1:1683))/1000;

%differentiate velocity data to get acceleration data

Ax=diff(Vx1)*framerate; %where framerate = 500.  If framerate is 1/500, use diff(x2)/framerate
Ay=diff(Vy1)*framerate;
Axy=sqrt((Ax.^2)+(Ay.^2));

%divide force data by mass to get acceleration data

Faccel = ha/0.830;


%aligning forces and position data to direction of strike 5 frames
%after strike has begun

%shift x and y axis to align with strike direction
xa = x2*cos(141)+y2*sin(141); %strike is 75 deg off horiz
ya = -x2*sin(141)+y2*cos(141); %%%WAS 141 for analysis, changed to 152 to test henry's hypoth.

%SMOOTH BEFORE CALCULATING
[sp,sxa]=spaps(time,xa,0.5); %0.5 looked good
[sp,sya]=spaps(time,ya,0.5); %0.5 looked good
```

Journal of Experimental Biology • Supplementary information

```
%differentiate position data to get velocity data

Vxa=diff(sxa)*framerate; %where framerate = 500.  If framerate is 1/500, use diff(x2)/framerate
Vya=diff(sya)*framerate;
Vxya=sqrt((Vxa.^2)+(Vya.^2));

%convert to m/s vs mm/s
Vxb=Vxa/1000;
Vyb=Vya/1000;
Vxyb=Vxya/1000;

fx2=fx1*cos(104)+fy1*sin(104); %was 345.8
fy2=-fx1*sin(104)+fy1*cos(104);
fz2=fz1*(-1);

fxyhyp = sqrt((fx2.^2)+(fy2.^2)+(fz1.^2));

fm=fx2;
fn=fy2;

%SMOOTH BEFORE CALCULATING
[sp,sfm]=spaps(timeb,fm,0.015); %0.5 looked good
[sp,sfn]=spaps(timeb,fn,0.015); %0.5 looked good

figure(1)
plot(timeb,fx2,'.','Color',[254,127,156]/255,'MarkerSize',5); %pink
hold on
plot(timeb,fy2,'.','Color',[135,206,235]/255,'MarkerSize',10); %light blue
plot(timeb,sfm,'.','Color',[255,0,0]/255,'MarkerSize',5); %red
plot(timeb,sfn,'.','Color',[0,0,255]/255,'MarkerSize',5); %blue

figure(2)
plot(time(31:304),Vxb,'.','Color',[255,0,0]/255,'MarkerSize',5); %red
```

Journal of Experimental Biology • Supplementary information

```
hold on;
plot(time(31:304),Vyb,'.','Color',[0,0,255]/255,'MarkerSize',5); %blue
plot(time(31:304),Vxyb,'.','Color',[75,0,130]/255,'MarkerSize',5); %purple
plot(timeb,zero2,'-','Color',[0,0,0]/255,'MarkerSize',5);
plot(timeb,fxyhyp,'-','Color',[100,100,100]/255,'MarkerSize',5);
plot(timeb,-sfn,'.','Color',[254,127,156]/255,'MarkerSize',5); %pink
plot(timeb,sfm,'.','Color',[135,206,235]/255,'MarkerSize',5); %light blue
plot(timeb,fz2,'.','Color',[192,192,192]/255,'MarkerSize',5); %dk gray

line([0.16834,0.16834],[-3,14],'LineStyle',':','Color',[0,0,0]/255,'MarkerSize',5); %snake straight
line([0.19246,0.19246],[-3,14],'LineStyle','--','Color',[0,0,0]/255,'MarkerSize',5); %forward progress ends
line([0.41156,0.41156],[-3,14],'Color',[0,0,0]/255,'MarkerSize',5); %snake lands on platform

% xlim([0,1]);
% ylim([-3,14]);

[pks,locs]=findpeaks(Vxy1);

dist = sqrt(((x2(84)-x2(30))^2)+((y2(84)-y2(30))^2));
dist1 = sqrt(((x2(96)-x2(30))^2)+((y2(96)-y2(30))^2));
angle = (y2(locs(2)+30)-(y2(30)))/(x2(locs(2)+30)-(x2(30)))*(180/pi);

%Tail Data
%Load data
xn = datan(:,[1]);
yn = datan(:,[2]);

%Adjust values to 'zero' them
xo = xn-xn(30);
yo = yn-yn(30);

%Convert pixels to mm
xp = xo/1.257;
yp = yo/1.257;
```

Journal of Experimental Biology • Supplementary information

```
%shift x and y axis to align with strike direction
xq = xp*cos(141)+yp*sin(141); %strike is 75 deg off horiz
yq = -xp*sin(141)+yp*cos(141);

%SMOOTH BEFORE CALCULATING
[sp,sxq]=spaps(time,xq,0.5); %0.5 looked good
[sp,syq]=spaps(time,yq,0.5); %0.5 looked good

%differentiate position data to get velocity data
Vxq=diff(sxq)*framerate; %where framerate = 500.  If framerate is 1/500, use diff(x2)/framerate
Vyq=diff(syq)*framerate;
Vxyq=sqrt((Vxq.^2)+(Vyq.^2));

%convert mm/s to m/s
Vxr=Vxq/1000;
Vyr=Vyq/1000;
Vxyr=Vxyq/1000;

figure(3)
plot(time(31:305),Vxr,'.','Color',[255,0,0]/255,'MarkerSize',5); %red
hold on;
plot(time(31:305),Vyr,'.','Color',[0,0,255]/255,'MarkerSize',5); %blue
plot(time(31:305),Vxyr,'.','Color',[0,255,0]/255,'MarkerSize',5); %green
plot(timeb,zero2,'-','Color',[0,0,0]/255,'MarkerSize',5);

line([0.16834,0.16834],[-3,14],'LineStyle',':','Color',[0,0,0]/255,'MarkerSize',5); %snake straight
line([0.19246,0.19246],[-3,14],'LineStyle','--','Color',[0,0,0]/255,'MarkerSize',5); %forward progress ends
line([0.41156,0.41156],[-3,14],'Color',[0,0,0]/255,'MarkerSize',5); %snake lands on platform

%differentiate tail velocity data to get tail acceleration data

Axt=diff(Vxr)*framerate; %where framerate = 500.  If framerate is 1/500, use diff(x2)/framerate
Ayt=diff(Vyr)*framerate;
```

Journal of Experimental Biology • Supplementary information

```
Axyt=sqrt((Axt.^2)+(Ayt.^2));

figure(4)
plot(time(32:305),Axt,'.','Color',[255,0,0]/255,'MarkerSize',5); %red
hold on;
plot(time(32:305),Ayt,'.','Color',[0,0,255]/255,'MarkerSize',5); %blue
plot(time(32:305),Axyt,'.','Color',[0,255,0]/255,'MarkerSize',5); %green
plot(timeb,zero2,'-','Color',[0,0,0]/255,'MarkerSize',5);

line([0.16834,0.16834],[-3,14],'LineStyle',':','Color',[0,0,0]/255,'MarkerSize',5); %snake straight
line([0.19246,0.19246],[-3,14],'LineStyle','--','Color',[0,0,0]/255,'MarkerSize',5); %forward progress ends
line([0.41156,0.41156],[-3,14],'Color',[0,0,0]/255,'MarkerSize',5); %snake lands on platform

[pkstailvel,locs]=findpeaks(Vxyr);
[pkstailacc,locs]=findpeaks(Axyt);

dist_tail = sqrt(((xq(114)-xq(30))^2)+((yq(114)-yq(30))^2)); %was 96

dist_tailx = sqrt((xq(114)-xq(30))^2); %was 124
dist_taily = sqrt(((yq(114)-yq(30))^2)); %was 124

MaxTailVely = max(Vyr);
MinTailVely = min(Vyr);

MaxTailVelx = max(Vxr);
MinTailVelx = min(Vxr);

MaxTailAccely = max(Ayt);
MinTailAccely = min(Ayt);

MaxTailAccelx = max(Axt);
MinTailAccelx = min(Axt);

%forces
```

Journal of Experimental Biology • Supplementary information

```
Fttl = sqrt((fx1.^2)+(fy1.^2)+((fz2+6.95).^2));
[sp,sft]=spaps(timea,fxyhyp,0.5); %0.5 looked good
figure(5)
plot(timeb,zero2,'Color',[0,0,0]/255,'LineWidth',1);
hold on;
plot(timeb,Fttl,'Color',[147,20,255]/255,'LineWidth',2); %ttl force-gray/purple was 216,191,216
plot(timeb,fz2+6.95,'Color',[192,192,192]/255,'LineWidth',2); %gravity force-gray
plot(timeb,-sfn,'Color',[255,0,0]/255,'LineWidth',2); %forward force-pink, was 254,127,156
plot(timeb,sfm,'Color',[0,0,255]/255,'LineWidth',2); %lateral force-light blue,135,206,235

line([0.16834,0.16834],[-5,16],'LineStyle',':','Color',[0,0,0]/255,'MarkerSize',5); %snake straight
line([0.19246,0.19246],[-5,16],'LineStyle','--','Color',[0,0,0]/255,'MarkerSize',5); %forward progress ends
line([0.41156,0.41156],[-5,16],'Color',[0,0,0]/255,'MarkerSize',5); %snake lands on platform
line([0,0.2],[5.24,5.24],'LineStyle','-.','Color',[0,0,0]/255,'MarkerSize',5); %body weight of snake in newtons


xlim([0.05,0.2]);
ylim([-5,16]);
ax=gca;
ax.FontSize=15;

%velocity
figure(6);
plot(time(31:304)+0.02,Vxb,'Color',[255,0,0]/255,'LineWidth',2); %forward vel-red
hold on;
plot(time(31:304)+0.02,Vyb,'Color',[0,0,255]/255,'LineWidth',2); %lat vel-blue, was 105,145,255
plot(time(31:304)+0.02,Vxyb,'Color',[147,20,255]/255,'LineWidth',2); %ttl vel-purple
plot(timeb,zero2,'-','Color',[0,0,0]/255,'MarkerSize',5);

% plot(time(31:304)+0.02,Vxr(1:274),'LineStyle',':','Color',[255,140,0]/255,'LineWidth',2); %tail dk orng
plot(time(31:304)+0.02,Vyr(1:274),'LineStyle','--','Color',[255,87,51]/255,'LineWidth',2); %tail light orng

line([0.16834,0.16834],[-3,4],'LineStyle',':','Color',[0,0,0]/255,'MarkerSize',5); %snake straight
line([0.19246,0.19246],[-3,4],'LineStyle','--','Color',[0,0,0]/255,'MarkerSize',5); %forward progress ends
```

Journal of Experimental Biology • Supplementary information

```
line([0.41156,0.41156],[-3,4],'Color',[0,0,0]/255,'MarkerSize',5); %snake lands on platform

xlim([0.05,0.2]);
ylim([-3,4]);
ax=gca;
ax.FontSize=15;

%acceleration
figure(7);
plot(time(32:304)+0.02,-Ax,'Color',[255,0,0]/255,'LineWidth',2); %red,was 220,20,60
hold on;
plot(time(32:304)+0.02,Ay,'Color',[0,0,255]/255,'LineWidth',2); %blue
plot(time(32:304)+0.02,Axy,'Color',[147,20,255]/255,'LineWidth',2); %purple was 75,0,130
plot(timeb,zero2,'-','Color',[0,0,0]/255,'MarkerSize',5);
% plot(timeb,Faccel,'-','Color',[192,192,192]/255,'MarkerSize',5);

% plot(time(32:304)+0.02,Axt(2:274),'LineStyle',':','Color',[255,140,0]/255,'LineWidth',2); %tail accel dk orng
plot(time(32:304)+0.02,Ayt(2:274),'LineStyle','--','Color',[255,87,51]/255,'LineWidth',2); %tail accel dk orng

line([0.16834,0.16834],[-300,300],'LineStyle',':','Color',[0,0,0]/255,'MarkerSize',5); %snake straight
line([0.19246,0.19246],[-300,300],'LineStyle','--','Color',[0,0,0]/255,'MarkerSize',5); %forward progress ends
% line([0.41156,0.41156],[-300,300],'Color',[0,0,0]/255,'MarkerSize',5); %snake lands on platform

xlim([0.05,0.2]);
ylim([-300,300]);
ax=gca;
ax.FontSize=15;

%Redo Force Measurements

%force impulse (fore-aft)
Foreaftimpulse = trapz(sfn(1:1683))/1000;

%max ttl force
```

Journal of Experimental Biology • Supplementary information

```
fz3 = fz2+6.95;
% fxyhypTtl = sqrt((fx2.^2)+(fy2.^2)+(fz3.^2));
fxyhypTtl = sqrt((fx2.^2)+(fy2.^2)+(fz3.^2));
[pksttlf,locs]=findpeaks(fxyhypTtl);

%max lateral force
[pkslatf,locs]=findpeaks(sfn);

%max vertical force
[pksvertf,locs]=findpeaks(fz3);

figure(8)
plot(timeb,zero2,'-','Color',[0,0,0]/255,'MarkerSize',5);
hold on;
plot(timeb,fxyhypTtl,'-','Color',[100,100,100]/255,'MarkerSize',5); %dkgray
plot(timeb,sfn,'.','Color',[135,206,235]/255,'MarkerSize',5); %light blue
plot(timeb,sfm,'.','Color',[254,127,156]/255,'MarkerSize',5); %pink
plot(timeb,fz3,'.','Color',[192,192,192]/255,'MarkerSize',5); %gray
% plot(timeb,fz2,'.','Color',[0,0,0]/255,'MarkerSize',5); % black


%Redo Fig. 5 with updated forces in BW
figure(9)
plot(timeb,zero2,'Color',[0,0,0]/255,'LineWidth',1);
hold on;
plot(timeb,Fttl/6.95,'Color',[147,20,255]/255,'LineWidth',2); %ttl force-gray/purple was 216,191,216
plot(timeb,(fz2+6.95)/6.95,'Color',[192,192,192]/255,'LineWidth',2); %gravity force-gray
plot(timeb,-sfn/6.95,'Color',[255,0,0]/255,'LineWidth',2); %forward force-pink, was 254,127,156
plot(timeb,sfm/6.95,'Color',[0,0,255]/255,'LineWidth',2); %lateral force-light blue,135,206,235

line([0.16834,0.16834],[-0.5,2.5],'LineStyle',':','Color',[0,0,0]/255,'MarkerSize',5); %snake straight
line([0.19246,0.19246],[-0.5,2.5],'LineStyle','--','Color',[0,0,0]/255,'MarkerSize',5); %forward progress ends
line([0.41156,0.41156],[-0.5,2.5],'Color',[0,0,0]/255,'MarkerSize',5); %snake lands on platform
line([0,0.2],[1,1],'LineStyle','-.','Color',[0,0,0]/255,'MarkerSize',5); %body weight of snake in newtons
```

Journal of Experimental Biology • Supplementary information

```
xlim([0.05,0.2]);
% ylim([-5,16]);
ax=gca;
ax.FontSize=15;

%Ratio test: fore-aft= -sfn; vertical= fz2
sfn1 = sfn/6.95;
fz21 = (fz2+6.95)/6.95;
fz22 = transpose(fz21);

Ratio = sfn1./fz22;

figure(15);
plot(timeb(1:7000),Ratio(1:7000),'Color',[152,29,151]/255,'LineWidth',1);
line([0,0.3],[0.3,0.3],'LineStyle',':','Color',[0,0,0]/255,'MarkerSize',5); %snake straight


xlim([0,0.3]);
ylim([-1,1]);
```
